# Reply: Teaching Anorectal Malformations

**Published:** 2015-07-01

**Authors:** Sushmita Bhatnagar

**Affiliations:** Department of Pediatric Surgery, BJ Wadia Hospital for Children, Mumbai

**Dear Sir**

The long term outcomes of anorectal malformations which include fecal incontinence, constipation, etc in both boys and girls is going to be covered in the Part 3 of the series of Anorectal Malformations. The continence mechanism anatomy and physiology is included therein. All the above topics as mentioned in your letter shall be covered in Part 3 which shall be published soon.

## Footnotes

**Source of Support:** Nil

**Conflict of Interest:** None

